# Ultra-high throughput sequencing-based small RNA discovery and discrete statistical biomarker analysis in a collection of cervical tumours and matched controls

**DOI:** 10.1186/1741-7007-8-58

**Published:** 2010-05-11

**Authors:** Daniela Witten, Robert Tibshirani, Sam Guoping Gu, Andrew Fire, Weng-Onn Lui

**Affiliations:** 1 Department of Statistics, Stanford University, Stanford, California 94305-4065, USA; 2 Department of Health Research and Policy, Stanford University, Stanford, California 94305-5405, USA; 3 Department of Pathology, Stanford University School of Medicine, Stanford, California 94305-5324, USA; 4 Department of Genetics, Stanford University School of Medicine, Stanford, California 94305-5324, USA; 5 Department of Molecular Medicine and Surgery, Karolinska University Hospital-Solna, Stockholm 17176, Sweden

## Abstract

**Background:**

Ultra-high throughput sequencing technologies provide opportunities both for discovery of novel molecular species and for detailed comparisons of gene expression patterns. Small RNA populations are particularly well suited to this analysis, as many different small RNAs can be completely sequenced in a single instrument run.

**Results:**

We prepared small RNA libraries from 29 tumour/normal pairs of human cervical tissue samples. Analysis of the resulting sequences (42 million in total) defined 64 new human microRNA (miRNA) genes. Both arms of the hairpin precursor were observed in twenty-three of the newly identified miRNA candidates. We tested several computational approaches for the analysis of class differences between high throughput sequencing datasets and describe a novel application of a log linear model that has provided the most effective analysis for this data. This method resulted in the identification of 67 miRNAs that were differentially-expressed between the tumour and normal samples at a false discovery rate less than 0.001.

**Conclusions:**

This approach can potentially be applied to any kind of RNA sequencing data for analysing differential sequence representation between biological sample sets.

## Background

Since the discovery that small RNA effectors define a number of developmental transitions and biological defence mechanisms [1,2], sequencing efforts in a variety of organisms have led to the recognition of several distinct small RNA sub-classes. These small RNAs (~18-30 nucleotides in length) function by guiding sequence-specific gene silencing at the transcriptional and/or post-transcriptional level and have been shown to play important regulatory roles in diverse biological processes [3-5]. Among the small RNA classes, microRNA (miRNA) is the most abundant class in mammals. Over the past 5 years, more than 8000 different miRNA genes have been identified in animals and plants (miRBase release version 12.0 [6]), and the number is expected to continue growing.

miRNA genes were first discovered by forward genetic methods. These methods led to the identification of several miRNA genes associated with developmental phenotypes in *Caenorhabditis elegans *(for example, *lin-4*, *let-7 *and *lsy-6*) [2,7-9] and programmed cell death in *Drosophila melanogaster *(for example, *miR-14 *and *bantam*) [10,11]. Forward genetics approaches are relatively inefficient for miRNA gene discovery, in part because of a small mutagenic target size and in part due to functional redundancy. The development of large-scale RNA sequencing methods [12-15] has greatly facilitated miRNA discovery, with thousands of miRNAs now identified from various cell lines and tissues from a variety of organisms. Apart from serving as a tool for novel small RNA discovery, the small RNA sequencing approach offers the potential to quantify and detect variation in mature miRNAs, including RNA editing [16-18] and 5'/3'-end variations [19-21]. Recent developments in ultra-high throughput sequencing technology greatly augment this approach, providing the possibility of a near-complete view of miRNA profiles.

Small RNA profiling by deep sequencing has been applied in an increasing variety of biological situations (for example, [22-31]). While greatly expanding the possibilities for precise expression profiling, sequencing-based profiling methods also raise new quantitative issues in recognizing and representing variation and significance in the resulting data sets. Many parallel questions were addressed in the early days of microarray analysis. Although sequence count data is analogous in some ways to microarray data, the two data types differ in numerous ways. First, microarray data provides an analogue measure of sequence prevalence while sequencing is inherently digital. Second, microarray analyses generally operate above a low background level of non-specific and off-target probe-array binding that can complicate the analysis of low-abundance molecular species (particular in cases where a related highly abundant product is present). With large enough sample sets, sequence-based analysis can avoid these background problems, allowing exquisite sensitivity. Still, rare molecular species are certainly subject to stochastic fluctuations in sequence data sets and these fluctuations can be large components of the total signal in cases where the counts of individual species are small. Microarray and sequence-counting based approaches share certain challenges, including biological and non-biological contamination and sample quality and reliability. Finally, it should be pointed out that microarray and sequencing procedures each give relative (and not absolute) measures of sequence abundance. Thus, the most informative comparisons look at changes in an expression ratio (involving at least two sequences) between two samples. This makes absolute comparisons of RNA abundance for different sequences problematic. Comparisons of relative RNA levels avoid such challenges and have been the focus of many analytical processes in both areas. In this work, we generate and analyse a large dataset of small RNA sequences in cervical cancer/normal sample pairs. We show that this approach provides an extensive coverage of miRNAs expressed in human cervical cancer tissues and normal cervices, including the detection of many previously known human miRNAs and their respective miRNA* sequences, as well as the identification of a number of novel miRNAs. Based on this sequence data we describe a statistical approach for cancer classification and we propose a new method for the identification of diagnostic miRNAs using sequencing-based miRNA profiling data. This approach should have general utility in analysing differential sequence representation between biological sample sets.

## Results

### miRNA profiling by small RNA cDNA library sequencing

We captured, amplified and sequenced 58 small RNA libraries prepared from 29 pairs of cervical cancer tissues and matched normal tissues (Additional File 1). In addition, the capture, amplification and sequencing for two small RNA libraries (G699N and G761T) were repeated in order to determine the reproducibility of the results. A total of 42,348,326 independent small RNA sequences (25,007,613 from normal cervices and 17,340,713 from cervical cancer samples) were obtained (Additional File 1). The average library coverage was 705,805 sequences (ranging from 29,848 to 2,624,426 for individual libraries) with the sequenced population containing 32.4% (13,710,440) miRNA sequences representing 626 distinct mature miRNAs (Additional File 2). Of these, 210 miRNA genes produced sequencing reads corresponding to both arms of the miRNA precursors. As expected, a majority of miRNA genes displayed strand bias. The relative abundance of most of the star forms (miRNA*) was lower than that of their corresponding miRNAs. However, six miRNAs (*miR-17*, *miR-202*, *miR-425*, *miR-493*, *miR-624 *and *miR-625*) had a higher number of sequencing reads originating from the annotated miRNA* strand than the mature miRNA sequence across majority of the libraries (Additional File 3). Some miRNA genes demonstrated a nearly equal number of sequencing reads originating from the 5' and 3' arms of the miRNA precursor.

The sequence data reveal a very broad range of expression levels for known miRNAs (based on sequence counts): ~6% of miRNAs were detected at high sequence counts (>10^4^), 14% were in the intermediate range (10^3^-10^4^), and the remaining were at low sequence counts (<100) (Additional File 3).

### Novel miRNA genes

To search for novel candidate miRNAs, we used criteria as previously described [32]: (i) at least 20 consecutive bases (measured from the start of the small RNA) aligned to human genome without any gaps; (ii) formation of a sufficiently low-energy (<-20 kcal/mol) secondary fold-back hairpin structure with small internal bulge(s) within the miRNA region and (iii) complete containment of the cDNA sequence within one arm of a hairpin. The resulting set of hairpin-derived small RNAs was further analysed to distinguish genuine miRNA precursors from other RNAs with similar structures.

Sixty-four novel miRNA genes (88 distinct mature miRNAs) were identified from a total of 45,299 novel sequence reads (Additional File 4). Twenty-three of these newly identified miRNA candidates were represented both in 5' and 3' arms of the hairpin precursor, providing strong evidence for biogenesis from a hairpin precursor. Two of the miRNA candidates were classified as mirtrons (intronic miRNA precursors that bypass Drosha processing) [33-35]. A distinct characteristic of a mirtron is that the miRNA precursor is directly adjacent to the splice sites. Among the novel candidate miRNAs, we identified eight putative antisense miRNAs (referring to those miRNAs derived from the antisense strand of annotated miRNA genes). Seven of these are antisense to known miRNA genes, while the eighth is antisense to a novel miRNA identified in this study (*miR-3622a *and *miR-3622b*). All new miRNAs (except one, *miR-1323-3p*) were observed more than once and detected in more than one library (Additional File 2). Although the majority of the newly identified miRNAs was detected at low abundance (as reflected by low sequence count across all the libraries), some were rather prominent (Additional File 2). Among these, *miR-1246 *was the most abundant with >13,000 sequencing reads detected.

Forty-one of the new miRNAs were located in introns, one in an exon, and five in the 5'/3' untranslated regions of known genes; 17 were found in the intergenic regions. Notably, *miR-3608 *is located adjacent to a vault RNA, *HVG-2 *(Additional File 5). Vault RNAs are a noncoding RNA family as part of the vault ribonucleoprotein complex that has been suggested to be involved in multidrug resistance [36]. Interestingly, this candidate was only detected in cervical cancer samples (G547T, G659T and G026T) (Additional File 2).

### miRNA data analysis

Sequencing-based miRNA profiling does not provide absolute measurements of miRNA expression, but rather the relative counts of different miRNAs within each sample. As described in the previous section, miRNA-seq data are typically characterized by variances in total counts for each sample. These, as well as sequence counts for individual miRNAs, will be subject to large sampling noise. Moreover, in contrast to microarray data, the miRNA-seq data involve non-negative counts.

All statistical analyses were performed on the cube-rooted data, unless otherwise specified. The raw data had a very skewed distribution, with many large values.

The cube-root transformation reduced this skewness and gave the resultant data an approximate Poisson distribution, which was important for our log-linear modelling. The standard approach for testing differential expression of genes measured on microarrays is to compute a *t*-statistic for each gene; a permutation distribution is then used to estimate false discovery rates. The use of a *t*-statistic is justified if the data are approximately normally distributed with equal variances, as is the case for microarray data after suitable transformations. However, since sequencing data involve non-negative counts, the assumption of normality is not appropriate. We instead develop a new method to identify differentially-expressed sequences based on a Poisson log linear model.

In order to evaluate reproducibility between replicates, we prepared two additional (duplicate) libraries for which small RNA capture, amplification and sequencing were carried out independently (and at a different time) from the respective original samples. The two samples for which this was done were G699N and G761T. From the raw data, we saw correlations of 0.8966 between the two libraries from G699N and 0.7836 for the two libraries from G761T. For reference, the mean correlation between pairs of different normal tissue samples was 0.6708, with the mean correlation between pairs of samples from different tumours being 0.5735. The observed duplicates are by no means perfectly concordant between replicate samples; in addition, we noted that some non-replicate pairs show more correlation with each other than do the pairs of replicates.

**Figure 1:**
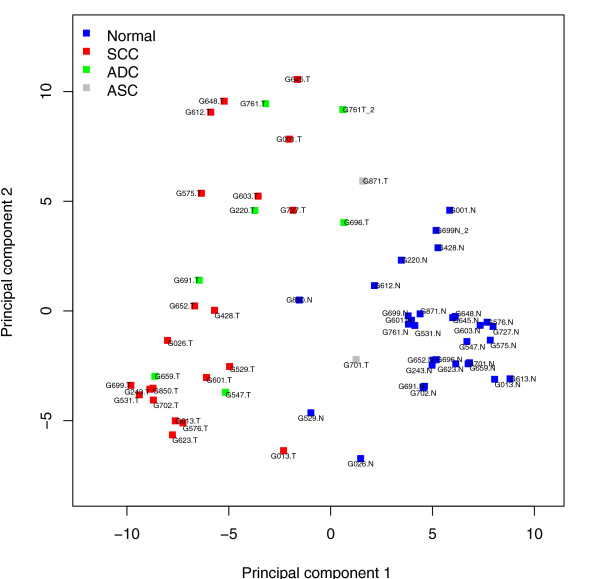
**Distinctive patterns of miRNA expression between cervical cancer and normal samples revealed by principal component analysis**. microRNA incidence values from each sample were projected onto the first two principal components, using cube-rooted data. This two-dimensional representation of the ~714 dimensional primary data resulted in evident separation between normal and tumour samples but not between adenocarcinoma (ADC) and squamous cell carcinoma (SCC) samples. The first principal component explains 21.2% of the variation present in the data and the second explains 11.6%. ASC, adenosquamous cell carcinoma; T, tumour; N, normal.

In order to visualize the 714-dimensional vectors of miRNA expression in a lower-dimensional subspace, we performed principal components analysis (PCA). The principal components are the linear combinations of the miRNAs that have the largest variance and provide informative axes for projection of the data. The analysis revealed clear separation between tumour and normal samples, but not between tumour types (Figure 1).

**Figure 2:**
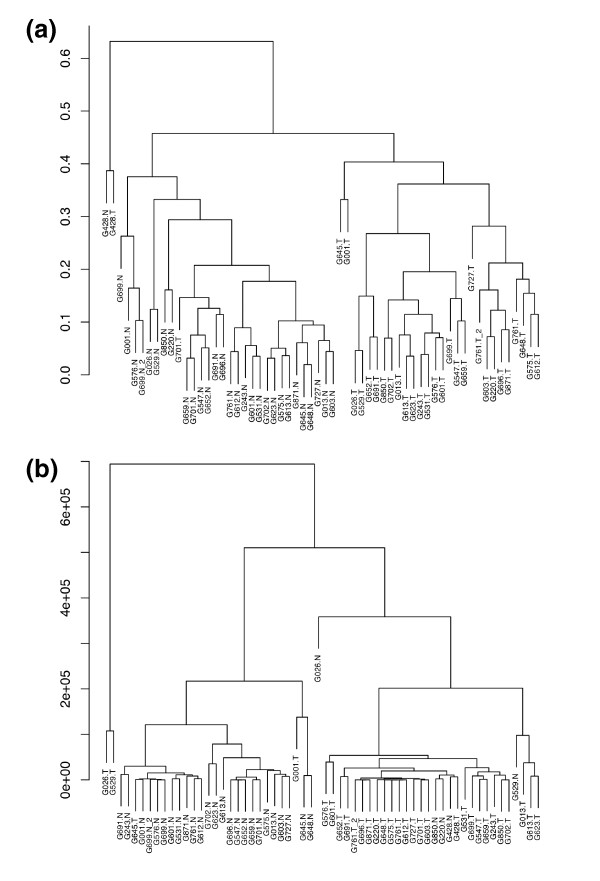
**Clustering analyses of normal and tumour samples based on microRNA expression. (a) Samples were clustered using cube-rooted data and correlation-based distance (as described in **Additional File 9). Two large subgroups and one small outgroup resulted, with separations 1N:1T, 29N:1T and 0N:28T, respectively. The small outgroup consisted of tumour and normal samples from patient G428. The remaining samples were partitioned among the two larger subgroups, one of which consisted of the other 29 normal samples and one tumour sample, and the other consisted of the remaining 28 tumour samples. (b) Samples were clustered using the distance metric defined in Section 4.1 of Berninger *et al*. [37]. Again, an outgroup and two major subgroups resulted, with separations 0N:2T, 25N:2T and 5N:26T, respectively. For both panels, 'N' indicates a normal sample and 'T' indicates a tumour sample. Note: the duplicates of G699N and G761T are clustered near each other in both methods.

In order to assess the difference between normal and tumour samples, we performed an unsupervised hierarchical clustering of the samples using complete linkage and correlation-based distance. Hierarchical clustering groups the samples by their similarity, in a bottom-up fashion. As shown in Figure 2A, the clustering analysis resulted in the identification of two major subgroups that show an almost perfect separation between normal and tumour samples. Recently, Berninger *et al*. also presented a method for defining distances between samples for miRNA expression profiling based on small RNA cloning data [37]. For comparison, we also performed clustering using the distance measure defined in Berninger *et al*. and the results revealed two subgroups with good separation (Figure 2B).

In order to classify samples based on miRNA expression levels, we applied the nearest shrunken centroids (NSC) method [38]. This method classifies samples by computing an average miRNA expression vector for each class; these average expression vectors are then shrunken towards the overall miRNA expression mean across the classes in order to avoid over-fitting and to obtain a classifier that makes use of only a subset of the miRNAs. Cross-validation (CV), a process in which samples are repeatedly split into training and test sets, was performed in order to select the optimal number of miRNAs to use in the classifier and to assess its accuracy. Applying NSC for: (i) normal versus tumour resulted in 4/58 CV errors (*P *< 0.002); (ii) normal versus adenocarcinoma (ADC) versus squamous cell carcinoma (SCC) resulted in 7/56 CV errors (*P *< 0.002); and (iii) ADC versus SCC resulted in 4/27 CV errors (*P *= 0.064). The two adenosquamous carcinoma (ASC) samples were excluded from analyses (ii) and (iii). The miRNAs used in the three NSC classifiers are shown in Additional Files 6, 7, 8.

In order to further explore the performance of NSC for normal versus tumour samples, we randomly split the samples into a training set of 40 samples and a test set of 18 samples. We trained NSC on the training set and tested on the test set; this was repeated 100 times. This resulted in an average of 1.77 errors for normal versus tumour classification. The samples that were most frequently misclassified were G529N, G696T, G701T, G850N and G871T. Not surprisingly, the samples are located near the boundary of the tumour and normal samples in the principal component analysis (PCA) plot (Figure 1).

**Figure 3:**
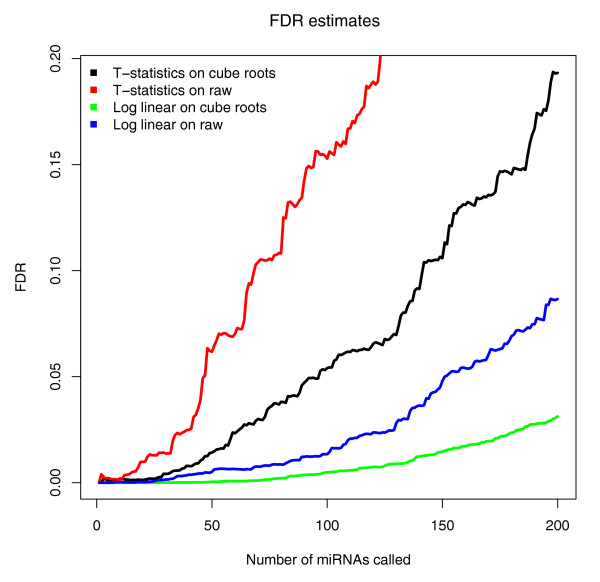
**Comparison of false discovery rate estimates based on different statistical methods**. False discovery rates are shown for our proposed method for identification of significant microRNAs (miRNAs) - a log-linear model on cube-rooted data - as well as three competing methods: a log-linear model on raw data, *t*-statistics on raw data, and *t*-statistics on cube-rooted data. The log-linear model on cube-rooted data results in extremely low false discovery rates (FDRs). The FDR for a given miRNA score cutoff is the average proportion of miRNAs with scores above that cutoff that are 'false positives'; see Additional File 9 for details on FDR calculation.

In order to identify miRNAs that are differentially expressed between tumour and normal tissue, we needed to address the fact that the data were characterized by a high variance in sequence counts between samples as well as between miRNAs, and the fact that the data were discrete. For this purpose, we propose the use of a Poisson log-linear model. In this model, the cube-rooted counts for each miRNA for each sample are taken as Poisson random variables and the logs of the means of these Poisson variables are estimated using a linear model (see Additional File 9). We allow a separate term for each miRNA (since different miRNAs have different frequencies) and for each sample (since some samples have much higher counts of all miRNAs). An additional term for each miRNA quantifies the extent to which each miRNA's counts differ between tumour and normal tissue. That is, we model 1 + *X_ij _*~ *Poisson*(*μ_ij_*), where *X *denotes the cube-rooted data matrix, *i*indexes the miRNAs, *j *indexes the samples, and log(*μ_ij_*) = *β_i _*+ *γ_j _*+ *ρ_i_*(1_*j *∈ *Tumour *_-1_*j *∈ *Normal*_). Here, we are using indicator variable notation: 1_A _equals 1 if A is true and 0 otherwise. In order to test how well the model fits the data, we binned the observations based on their fitted mean value in the Poisson model and estimated the mean and variance of the observations in each bin. As expected, under the Poisson model, the mean and variance of the observations within each bin are approximately equal (Additional File 9). In our model,  can be thought of as a score for the extent to which miRNA *i *is differentially expressed between tumour and normal samples. Here, the denominator *se *(*ρ_i_*) indicates the standard error of *ρ_i_*. In order to estimate the false discovery rates (FDRs) for these scores, we randomly permuted tumour and normal sample labels and compared the observed  scores to the null distribution of these scores obtained by permutations. For comparisons, we also computed FDRs resulting from the log-linear model on raw data, *t*-statistics on raw data, and t-statistics on cube-rooted data. We found that our log-linear model on cube-rooted data resulted in extremely low FDRs (Figure 3). Based on the permutation results, only ~4 of the 200 miRNAs with highest estimated absolute  are false positives. The miRNAs with highest absolute  scores are shown in Table 1. (A list of all miRNAs with estimated absolute  scores and FDRs is available in Additional File 10). All computations were carried out using the *R *statistical package version 2.6.2.

**Table 1:** miRNAs with significant changes determined by our Poisson log-linear model

miRNA	****^†^	FDR^§^
*miR-205*	10.8525	0

*miR-143*	-8.292	0

miR-10b*	-7.6723	0

*miR-31*	6.7684	0

*miR-203*	6.4009	0

miR-145*	-6.2416	0

*miR-944*	6.1095	0

*miR-1*	-5.9363	0

*miR-1246*	5.7935	0

*miR-204*	-5.7914	0

*miR-1303-3p*	5.7469	0

miR-31*	-5.6673	0

miR-126*	-5.6346	0

*miR-7*	5.4282	0

*miR-10b*	-5.3186	0

*miR-425*	5.2653	0

miR-200a*	5.1818	0

*miR-125b*	-5.1426	0

*miR-140-5p*	-5.0996	0

miR-21*	5.0897	0

*miR-126*	-5.0092	0

miR-183*	4.9398	0

*miR-147b*	4.9109	0

*miR-155*	4.7346	0

miR-25*	4.6551	0

*miR-450a*	-4.584	0

*miR-142-3p*	4.5778	0

*miR-99b*	-4.427	0

*miR-424*	-4.3806	0

miR-141*	4.3352	0.0001

*miR-96*	4.3028	0.0001

*miR-3614-5p*	4.2005	0.0001

^† ^can be considered as a score for whether microRNA *i *is differentially expressed between tumour and normal: a large positive value indicates higher expression in tumour than normal.

^§ ^Only microRNAs with low FDR (≤ 0.0001) are shown.

FDR = false discovery rate.

### Dependence of analysis on sequencing scale

Variation in overall sequence depth for different samples will be present in any analysis of independent biological specimens due to differences in tissue makeup and abundance, with some additional variation due to technical aspects of library construction and sequencing. Our discussions above make use of datasets with numbers of sequence reads for the different samples that range from tens-of-thousands to over a million (Additional File 1). In order to characterize the effect of sequencing scale on the statistical results obtained, we generated resampled data sets with various numbers of miRNAs. That is, for *n *= 10^3^,10^4^,10^5 ^and 10^6^, we sampled with replacement *n *miRNAs from each of the 58 samples. We fit an NSC classifier in order to distinguish between normal and tumour samples using the resampled data sets. Regardless of the value of *n *in this range, around 4/58 cross-validation errors resulted. This suggests that the extent of library coverage does not greatly affect the classifier. We also computed the number of miRNAs found to be differentially-expressed at a given FDR threshold for each of the resampled data sets. The results can be seen in Additional File 11. As *n *increases, so does the number of miRNAs (as expected, particularly with a stringent FDR threshold). As *n *becomes quite large, the benefit of further increasing *n *in terms of new miRNA identification becomes marginal.

These results indicate that conclusions can be drawn at a variety of experimental scales, with deeper library coverage resulting in more power for some statistical analyses but with biological factors (for example, number and uniformity of specimens) eventually exerting the major limitations on interpretation as sequencing depth increases.

### Analysis of clustered miRNA expression

We defined a miRNA cluster as a set of miRNAs located (i) within a 1 kb region or (ii) in close proximity (<4 kb), with the same orientation and not separated by a miRNA in the opposite orientation. Our data contained 56 miRNA clusters consisting of 236 miRNAs (Additional File 12). In order to determine whether the clustered miRNAs showed significant co-expression, we calculated the average correlation of miRNA pairs within each cluster. As shown in Additional File 12, we observed that many clusters contain miRNAs that have more correlated expression than one would expect due to chance. Twenty-three miRNA clusters show significantly correlated expression (*P *< 0.05). Of these, eight clusters are highly correlated (*P *< 0.001). These include *miR-200b~429*, *miR-34b~34c*, *miR-503~424*, *miR-29c~29b*, *miR-15b~16*, *miR-200c~141, miR-99b~125a *and *miR-25~106b *clusters.

In order to determine whether any of the 56 clusters contain miRNAs that are significantly associated with the cervical cancer versus normal class labels, we applied the log linear model and calculated the median scores for the expressed miRNAs in each cluster. We found that 30 clusters contain expressed miRNAs that are significantly associated with the disease (*P *< 0.05; Additional File 13). Seventeen of the 30 clusters are associated with increased expression in cervical cancer and the remaining 13 clusters are associated with reduced expression in cancer. Interestingly, the two clusters that are most associated with the cervical cancer versus normal class labels both belong to the *miR-200 *family.

### Assessment of miRNA expression patterns

We compared the expression level of *miR-21 *and *miR-143 *in all cervical cancer samples and matched normal cervices used in this deep sequencing experiment with previously published Northern blot results of the same materials [32]. As shown in Additional File 14, both *miR-21 *and *miR-143 *expression patterns obtained from the sequencing data correlate well with the Northern data. Modest differences, observed for a number of the samples, could be indicative of non-linearity in either assay; alternatively such differences may be due to RNA cross-hybridization (an ability to pick up alternate miRNAs with the same probe), uncertainty in loading controls (in some cases the ribosomal bands are difficult to quantitate due to low levels) and to gel exposure artifacts (some Northern bands are difficult to distinguish from optical noise on the filters.).

## Discussion

In this work, we used high throughput sequencing approaches combined with statistical analysis in order to comprehensively characterize miRNA expression profiles of 29 matched pairs of human cervical cancer and normal cervical samples. Our results reveal a large number of miRNAs detected in all libraries and provide quantitative measures for a broad range of miRNA expression levels.

### miR* and antisense miR sequences

One aspect of miRNA diversity comes from the ability to produce two distinct miRNAs (termed miR-X and miR-X*) from a given hairpin precursor RNA. In this case, the two RNAs are distinct products of the same initial processing product (pre-RNA), one located 5' and one 3' on this precursor. The standard nomenclature for miRNAs assigns the asterisk to the less abundant of the two forms found in the first identifying study. In this work, we detected a large number of miR* sequences, as well as mature miRNAs from both 5' and 3' arms of the hairpin precursor. For six miRNAs, we detected a higher number of miR* sequence than the annotated mature miRNA sequence in a majority of the libraries. This may indicate that both 5' and 3' arms of the pre-miRNA can be expressed in specific tissues/cells and that they may have a functional relevance. Consistent with such a dual role, reversals of abundance between miR and miR* have been observed in several recent miRNA transcriptome analyses [20,25,39] and miR/miR* strand selection have also been shown to be different among different Argonaute complexes [40,41]. Furthermore, Okamura *et al*. recently demonstrated that some miRNA* species can associate with the RNA-induced silencing complex and have inhibitory function [42]. These findings suggest that there are additional levels of complexity in miRNA processing which remain to be determined.

Three recent studies demonstrate that sense and antisense miRNAs can be generated by bidirectional transcription of the *Drosophila *Hox miRNA locus *miR-iab-4 *[43-45]. Interestingly, these sense and antisense miRNAs are expressed in non-overlapping spatial domains and have different targets. This phenomenon is not restricted to the Hox loci in flies. Many more sense-antisense miRNA pairs have also been identified in flies and mammals [44,45]. Here, we provide further evidence for the existence of sense-antisense miRNA pairs in human tissues. Although these antisense miRNAs are low in abundance (<100 copies in all libraries), their low concentration does not rule out their possible biological relevance. Further investigations are warranted in order to assess the biological significance of these sense-antisense miRNA pairs for a complete understanding of the complexity of gene regulation by miRNAs.

### miRNA complexity

Given that our small RNA libraries were prepared from a single tissue type (cervical tissues), we unexpectedly found a large number of miRNAs (ranging from 156 to 555) in each library, with the number depending on the depth of sequencing. The data suggest that many miRNAs may lack complete tissue specificity, instead show a continuum of expression variability in different tissues/cells and physiological/pathological states. Alternatively, a small population of distinct cell types may be present in the samples used for the analysis and contribute to the low-level detection of large numbers of miRNAs. Data with cell lines, which have more homogenous cell populations, are of relevance to this issue. Consistent with the hypothesis of extensive diversity in a single cell type, Friedländer *et al*. recently detected a total of 213 known miRNAs in a single HeLa sample [46].

### miRNA expression in cervical cancer and matched controls

We used two statistical techniques to develop an understanding of the differences in miRNA expression between normal and tumour cervical tissue.

First, we used NSC [38], a method originally developed for microarray data analysis, to construct a classifier that performs cancer class prediction from sequencing-based miRNA expression profiling. The method successfully classified the normal and tumour samples in approximately 16 of 18 test samples. Second, in order to identify miRNAs that are differentially expressed between tumour and normal tissue, we developed a simple log linear model for data from ultra-high throughput sequencing. This model is analogous to using a *t*-statistic to identify differentially expressed genes in the case of microarray data. Unlike the *t*-statistic, it is appropriate in cases where the observations take on discrete values and where variation occurs between samples as well as between genes or miRNAs. This model resulted in the identification of a set of miRNAs that distinguish tumour from normal samples, with low FDRs (≤ 0.0001; Table 1 and Additional File 10). The model can potentially be applied to any kind of sequencing data that produce count data. Software implementing this log linear model will be made freely available.

In agreement with our [32] and other previous findings [47,48] with smaller data sets, the expression of *miR-143 *was significantly lower in cervical tumours as compared to their matched normal controls. Importantly, *miR-143 *has been shown to inhibit cell growth in HeLa cells [48], supporting its critical role in cervical carcinogenesis. Also, a suppressor role of *miR-143 *has also been implicated in different tumour types [49-51].

Among the most abundant miRNAs in the cervical cancer tissues, *miR-205 *has the highest estimated  score in the log linear model. Its increased expression has also been observed in a variety of carcinomas, including cervical cancer [48], endometrioid endometrial adenocarcinoma [52], ovarian cancer [53], bladder cancer [54], head and neck squamous cell carcinoma [55] and non-small cell lung cancer [56]. Very recently, Yu *et al*. demonstrated that the lipid phosphatase *SHIP2 *(SH-2 containing inositol 5'-phosphatase 2) can serve as a target of *miR-205 *in SCC cells [57], with down-regulation of *miR-205 *in SCC cells leading to a marked increase in apoptosis and cell death [57]. This will certainly provide an important lead in investigating roles for *miR-205 *in cervical cancer.

An additional miRNA demonstrating strong regulation, *miR-944 *(identified from small numbers of sequences in an earlier study; [32]), was significantly more abundant in the cervical cancer tissues than in their normal counterparts. This miRNA seems to be cervical tissue specific in that it had not been previously observed in other tissues or cell types [20]. *miR-944 *is located in the intron of *TP63 *(a member of the p53 family) and maps to chromosome 3q27-28, a region frequently amplified in cervical carcinomas [58-60]. It will be of interest to test for potential roles of *miR-944 *in cervical carcinogenesis and/or progression.

### Clustered miRNA expression

An analysis of clustered miRNA expression revealed strong positive correlations among the closely neighbouring miRNAs, suggesting that these miRNAs may be controlled by common regulatory factor(s). The data are in consistent with several previous findings [61-63]. Interestingly, we found that the *miR-200 *family of miRNAs (*miR-200a/b/c*, *miR-141*, and *miR-429*) was highly co-expressed in cervical cancer. These miRNAs are located at two different genomic loci: the *miR-200b~429 *cluster is located on chromosome 1, and the *miR-200c~141 *cluster is located on chromosome 12. The co-expression of these *miR-200 *loci suggests that these miRNA clusters might be co-regulated by common regulator(s) and function together. In line with such hypothesis, Bracken *et al*. recently demonstrated that both *miR-200b~429 *and *miR-200c~141 *clusters are encoded by single polycistronic primary miRNA transcripts [64]. Furthermore, the *E-cadherin *transcriptional repressor *ZEB1 *was found to directly suppress transcription of both clusters [64,65]. As the five *miR-200 *family members contain very similar seed sequences, these miRNAs are likely to regulate some common targets. In support, several independent studies showed that two transcriptional repressors of *E-cadherin*, *ZEB1 *and *ZEB2*, are the direct targets of the *miR-200 *family miRNAs [66-68]. Although the expression of *miR-200 *clusters is reduced in mesenchymal and invasive cells, its over-expression has also been observed in ovarian [69] and cervical [70] cancers.

### A unique small RNA downstream of the Vault transcript

Among the novel miRNAs discovered here, *miR-3608 *has the unique feature of sitting immediately downstream of vault RNA, *HVG-2 *(Additional File 5). Vault RNAs are small non-coding RNAs produced by RNA polymerase III [71]. The possibility that *miR-3608 *might be produced from the *HVG-2 *promoter suggests a type of dicistronic heterologous Pol III transcript similar to tRNA-miRNA dicistronic transcripts that have been identified in the mouse gammaherpesvirus 68 [72] and in the C19MC cluster of the human genome [73].

## Conclusions

Our approach illustrates the high value of ultra-high throughput sequencing data for novel miRNA discovery and quantitative analysis of miRNAs. The statistical approach described in this study is broadly applicable to the analysis of any RNA sequencing data.

## Methods

### Clinical samples

Twenty-nine pairs of snap-frozen cervical tumour and matched normal tissue were obtained from the Gynecologic Oncology Group Tissue Bank (PA, USA). Of these 29 cases with paired specimens, 21 patients had a diagnosis of SCC, six had ADC and two had an intermediate diagnosis of adenosquamous cell carcinoma (ASC) (Additional File 1). All matched normal cervical tissues were obtained from the same patients and had been histopathologically verified. This study was approved by the Institutional Review Board of Stanford University.

### Small RNA library construction and Solexa sequencing

Small RNA isolation was performed using mirVana miRNA isolation kit (Applied Biosystems/Ambion, TX, USA). The capture and amplification procedure was done as previously described [32], with slight modifications. Purified small RNAs were ligated to the 3'-adaptor ["Linker-1", IDT Inc., IA, USA] and 5' adaptor [5'-ACGCTCTTCCGATCT**v**-3' (uppercase, DNA; **v **= barcodes with triple RNA molecules: aaa, ggg, ccc or uuu; IDT Inc, IA, USA)] oligonucleotides. Products from the second ligation were gel-purified and reverse transcribed using the reverse transcription primer [5'-ATTGATGGTGCCTACAG-3']. cDNA was amplified with 16-20 polymerase chain reaction cycles, using a forward primer 5'-GAT ACG GCG ACC ACC GAG ATC TAC ACT CTT TCC CTA CAC GAC GCT CTT CCG ATC T-3' and a reverse primer 5'-CAA GCA GAA GAC GGC ATA CGA GCT CTT CCG ATC TAT TGA TGG TGC CTA CAG-3', to produce sequencing libraries that were subjected to Solexa/Illumina sequencing platform (Illumina 1G Genome Analyzer, CA, USA). Details of small RNA library preparation protocol are available upon request. The sequencing data have been deposited at Gene Expression Omnibus (accession No. GSE20592).

### Sequencing analysis

Individual sequence reads were initially generated following sequencing using the Solexa software pipeline (Illumina Inc, CA, USA). Reads from each of the pooled libraries were then separated based on their barcode sequence and mapped against human genome using ELAND (Solexa, Illumina Inc, CA, USA). Perfectly aligned sequences with at least 20 consecutive bases were analysed further. Aligned sequences were then further analysed with BLAST (blastn, [74]) and BLAT in order to exclude other known structural RNAs.

In order to identify sequence reads that match previously identified miRNAs, we aligned sequences against miRNA data from miRBase release version 10.1 [6] using BLAT [75]. miRNAs with varying 3' terminal were grouped together for tag counts. In order to uncover novel miRNA genes, we identified hairpin-like RNA structures in a window of 80 bases around recovered small RNA sequences using mfold (version 3.2 [76]). All predicted hairpin-like precursors were analysed carefully in order to distinguish genuine miRNA precursors from other RNA classes that may contain similar RNA structures (for example, snoRNAs, vault RNAs and tRNA-derived repeat elements).

### Statistical analysis

All statistical analyses were performed using the statistical software language R (version 2.6.2), freely available at http://cran.r-project.org/[77].

The miRNA count data are characterized by very large variances in both the total counts for each miRNA and the total counts for each sample. Total miRNA counts ranged from 1 to 2,253,073 (with a mean of 19,189), and total sample counts ranged from 1,322 to 1,227,057 (with a mean of 236,227). Because the row and column totals of the data matrix vary by many orders of magnitude, cube-rooted miRNA counts were used for almost all statistical analyses. Let *X *denote the matrix of cube-rooted data, where the rows denote the miRNAs and the columns denote the samples. In order to visualize the samples, we performed PCA after standardizing each column of *X *to have mean zero and standard deviation 1.

NSC [38] is a classification method intended for the case where the number of samples is small relative to the numbers of features or variables. A centroid (or mean vector) is computed for each class; the centroids are then 'shrunken' towards the overall centroid for the full data set. New observations are then classified to the shrunken centroid to which they are nearest. Depending on the amount of shrinkage performed, only a subset of the features will differ between the shrunken centroids. The number of features that differ between the shrunken centroids is treated as a tuning parameter for the method, and is selected by CV. NSC was performed using the R library 'pamr' on the cube-rooted data, after scaling each column of the cube-rooted data by the total for that column. NSC classifiers were constructed to distinguish between the following sets of classes: (i) tumour versus normal; (ii) tumour versus ADC versus SCC; and (iii) ADC versus SCC. For each classifier, the tuning parameter value (controlling the number of miRNAs used by the classifier) was selected by 10-fold CV. In order to obtain a *P*-value for each classifier, CV errors were computed on the real data and on null data obtained by randomly permuting the class labels for the samples. The *P*-value is given by the fraction of null data sets resulting in CV errors less than, or equal to, the CV error of the real data set. To further explore the performance of NSC for normal versus tumour, we randomly split the samples into a training set of 40 samples and a test set of 18 samples. We trained NSC on the training set and tested it on the test set; this was repeated 100 times. Only the first replicate in each pair was used in the NSC analysis.

We also took an unsupervised approach to assess the difference between normal and tumour samples: we used complete linkage and correlation-based distance to hierarchically cluster the cube-rooted data using the R language function 'hclust'. For comparison, we performed clustering using the distance metric defined in Berninger *et al*. [37].

In order to identify miRNAs that were differentially expressed between normal and tumour samples, we developed a Poisson log-linear model. The model assumes that 1 + *X_ij _*~ *Poisson*(*μ_ij_*) and log(*μ_ij_*) = *β_i _*+ *γ_j _*+ *ρ_i_*(1_*j *∈ *Tumour *_- 1_*j *∈ *Normal*_); that is, *β_i _*is the miRNA-specific term, *γ_j _*is the sample-specific term and *ρ_i _*is the difference between tumour and normal for miRNA *i*. We fit this model in two steps, using an offset. The quantity can be considered as a 'score' for whether miRNA *i *is differentially expressed between tumour and normal; a large positive value indicates higher expression in tumour than in normal. FDRs were estimated by permutations: tumour and normal sample labels were randomly permuted, and the estimated distributions of  for real and permuted data were compared. Details are given in Additional File 9. For comparison, we also computed FDRs resulting from our log-linear model using raw, rather than cube-rooted data, as well as FDRs resulting from computing a paired two-sample *t*-statistic for each miRNA (using both raw and cube-rooted data). Note that the first replicate from each pair of duplicate libraries was arbitrarily chosen for fitting the log linear model.

### Analysis of miRNA clusters

In this analysis, we considered any two miRNA precursors on the same chromosome strand (i) within 1 kb or (ii) within close proximity (<4 kb), but not separated by a miRNA in the opposite orientation as the same miRNA cluster. Using this cutoff, we identified 56 miRNA clusters, which contain 236 miRNAs from our datasets (Additional File 15).

In order to determine whether the miRNAs in cluster k have correlated expression, we computed the average correlation of miRNA pairs within the cluster. We estimated a null distribution for this average correlation by randomly sampling n_k _miRNAs from the full set of miRNAs and computing the average correlation of the pairs within this null cluster. The null distribution was used to estimate *P*-values for the extent to which the miRNAs in a single cluster are correlated with each other.

In order to determine whether the miRNAs in cluster k were significantly associated with the tumour/normal phenotype, we fitted the log linear model mentioned previously and computed the median of the resulting scores for the miRNAs in cluster k. We also permuted the tumour/normal labels repeatedly and each time re-fit the log linear model and recorded the resulting median miRNA score for cluster k. These median scores for the permuted data served as a null distribution, which we used to obtain a *P*-value for the extent to which each cluster k's miRNAs are associated with tumour/normal.

## Abbreviations

ADC: adenocarcinoma; SCC: squamous cell carcinoma; CV: cross validation; FDR: false discovery rate; miRNA: microRNA; NSC: nearest shrunken centroids; PCA: principal components analysis.

## Authors' contributions

WOL and AF conceived and designed the experiments. WOL performed the experiments. WOL, DW, RT, SG and AF analysed the data. WOL, DW, RT and AF contributed reagents/materials/analysis tools. WOL, DW, RT and AF wrote the manuscript. All authors have read and approved the final version of the manuscript.

## Supplementary Material

Additional file 1 Small RNA sequences obtained from 29 pairs of human cervical cancer tissues and matched normal tissues. Click here for file

Additional file 2 Known and novel microRNAs expressed in human cervical cancer tissues and matched normal tissues. Click here for file

Additional file 3 Distribution of sequence counts for number of unique microRNAs expressed in cervical cancer tissues and normal cervices. Click here for file

Additional file 4 Novel candidate microRNAs identified from human cervical cancer and normal cervices. Click here for file

Additional file 5 A unique small RNA downstream of the Vault transcript. Click here for file

Additional file 6 microRNAs (miRNAs) used in the nearest shrunken centroid classifier for normal versus tumour, as well as the standardized centroids for each of those miRNAs in each class. Click here for file

Additional file 7 microRNAs (miRNAs) used in the nearest shrunken centroid classifier for normal versus adenocarcinoma versus squamous cell carcinoma, as well as the standardized centroids for each of those miRNAs in each class. Click here for file

Additional file 8 microRNAs (miRNAs) used in the nearest shrunken centroid classifier for adenocarcinoma versus squamous cell carcinoma, as well as the standardized centroids for each of those miRNAs in each class. Click here for file

Additional file 9 Supplementary description of statistical analysis. Click here for file

Additional file 10 The false discovery rate of all microRNAs as determined by our Poisson log-linear model. Click here for file

Additional file 11 The number of microRNAs found to be differentially-expressed at a given false discovery rate threshold for each of the resampled data sets. Click here for file

Additional file 12 Average correlation of microRNAs within each cluster, and corresponding *P*-values. Click here for file

Additional file 13 Median score of microRNAs within each cluster in log linear model, and corresponding *P*-values. Click here for file

Additional file 14 Comparison of sequencing and Northern data of the 29 cervical cancer samples studied. Click here for file

Additional file 15 The correlation matrix for each microRNA cluster and its *P*-value. Click here for file
