# Supplementary figures and images for: Ultra-high throughput sequencing-based small RNA discovery and discrete statistical biomarker analysis in a collection of cervical tumours and matched controls
Source: BMC Biol. 2010 May 11;8:58. doi: 10.1186/1741-7007-8-58 (PMC2880020; doi:10.1186/1741-7007-8-58)

5q31.1

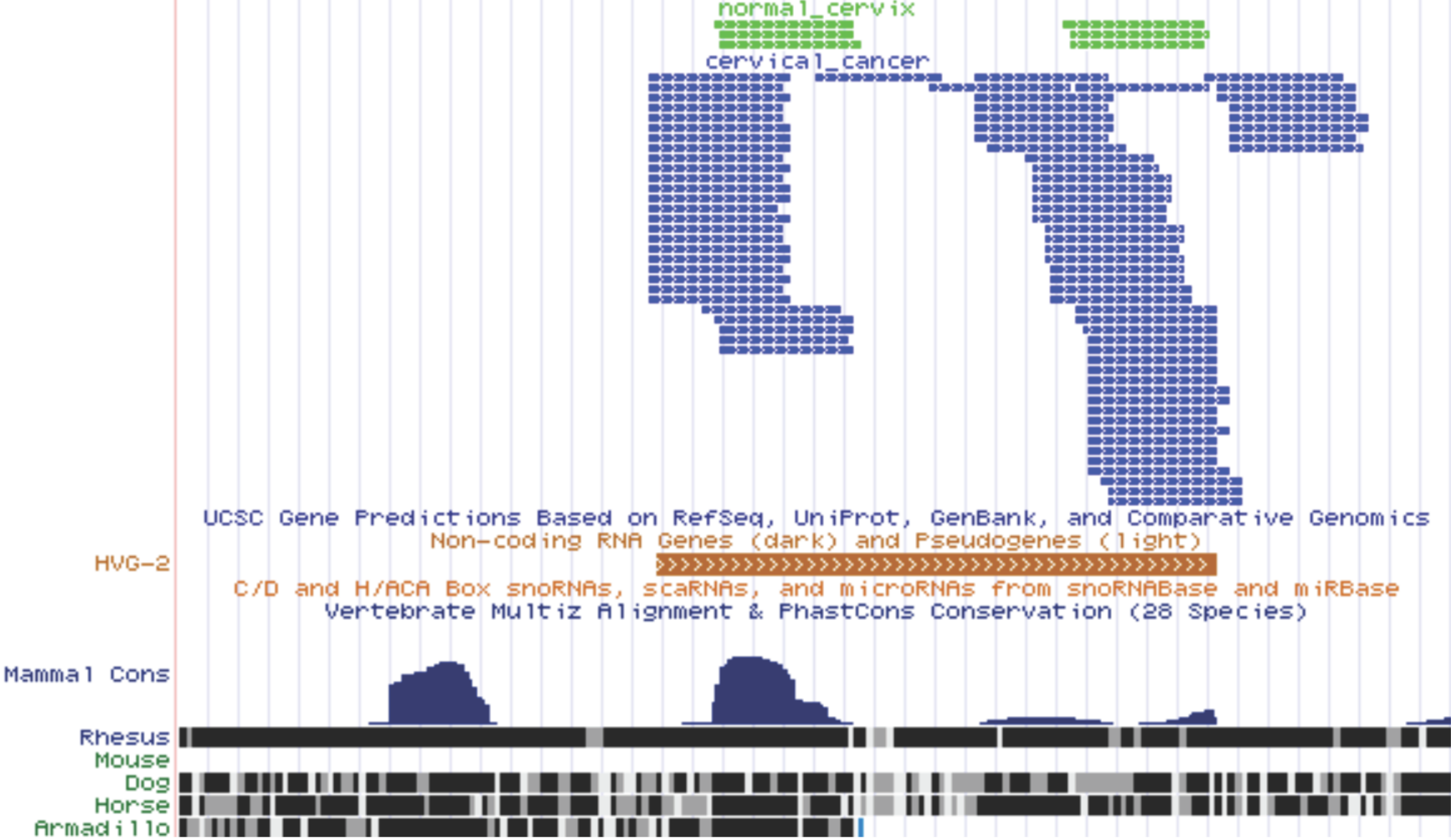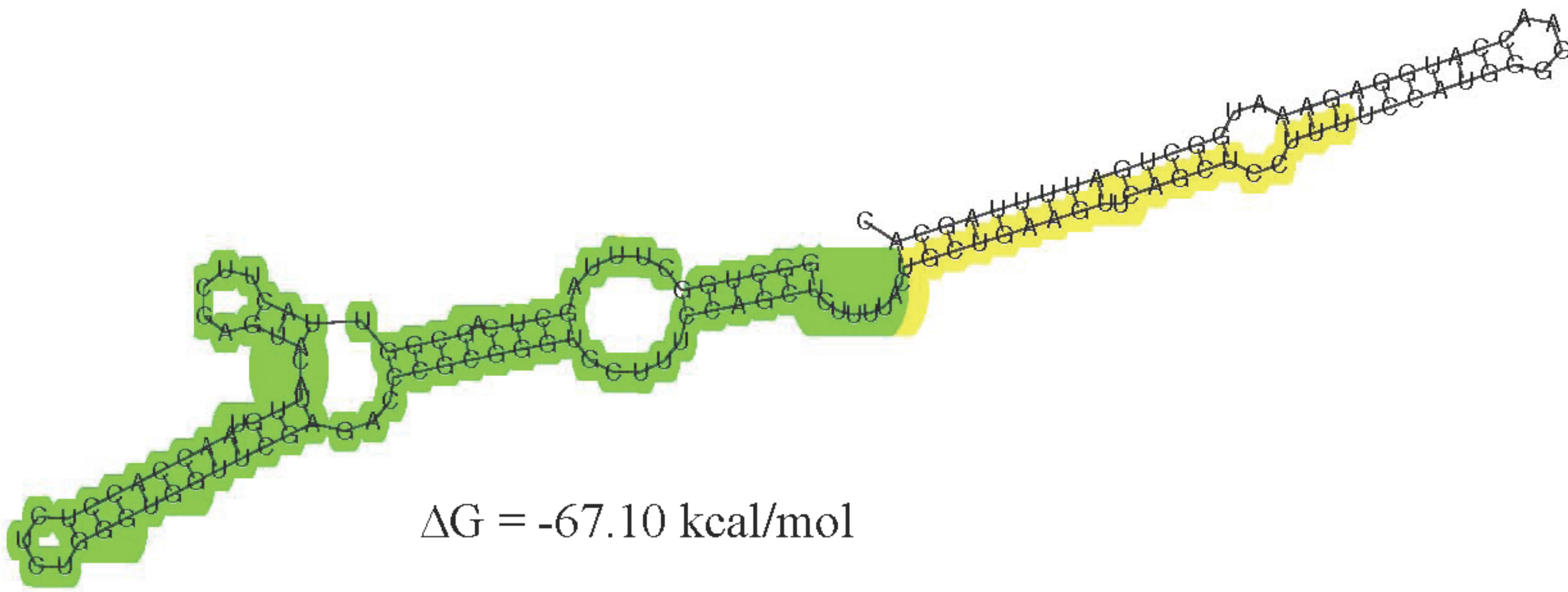

Supplement: Additional file 5 — A unique small RNA downstream of the Vault transcript. [file 1741-7007-8-58-S5.pdf]

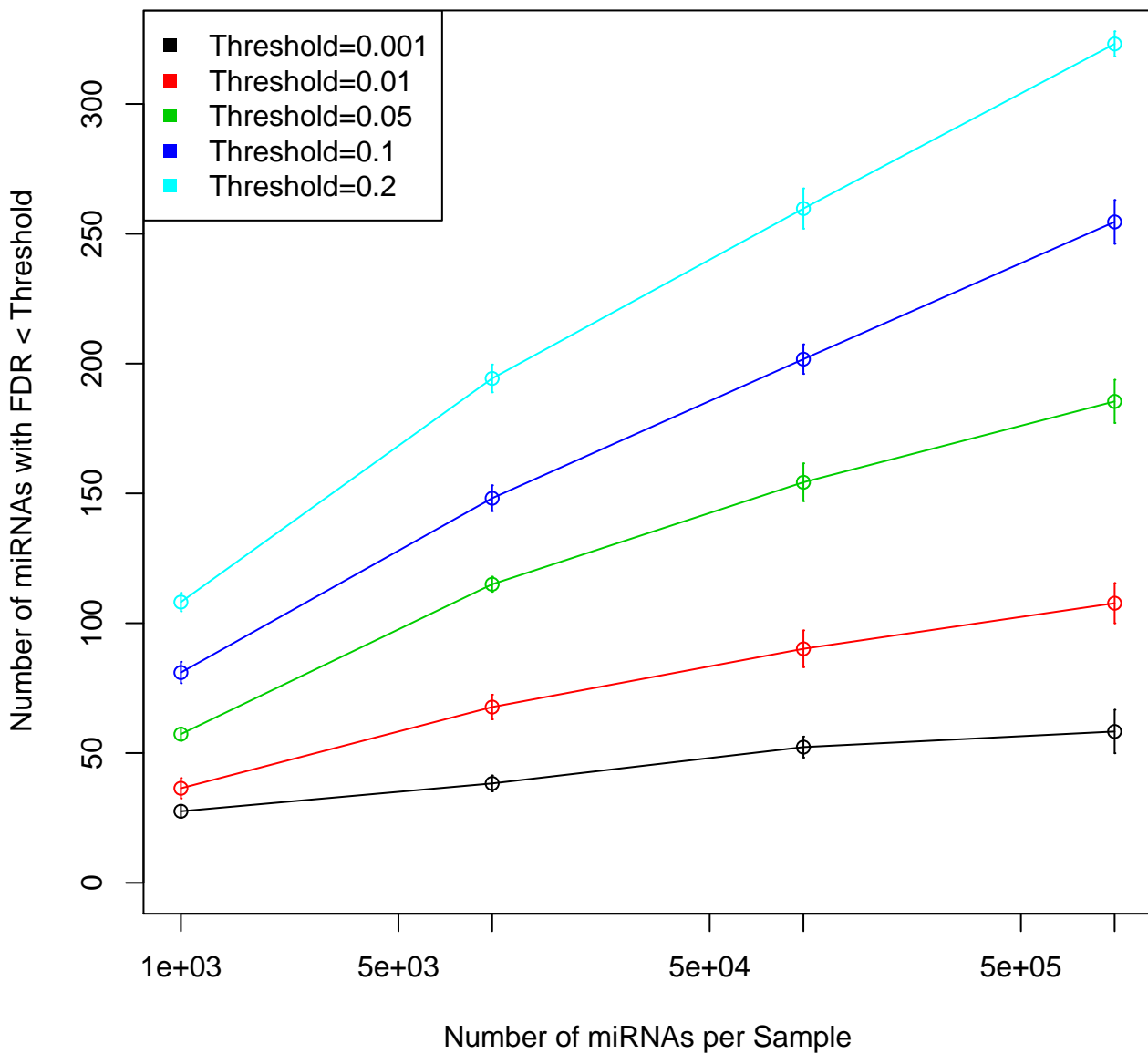

Supplement: Additional file 11 — The number of microRNAs found to be differentially-expressed at a given false discovery rate threshold for each of the resampled data sets. [file 1741-7007-8-58-S11.pdf]
