# Supplementary material for: Ultra-high throughput sequencing-based small RNA discovery and discrete statistical biomarker analysis in a collection of cervical tumours and matched controls
Source: BMC Biol. 2010 May 11;8:58. doi: 10.1186/1741-7007-8-58 (PMC2880020; doi:10.1186/1741-7007-8-58)
Supplement: Additional file 9 — Supplementary description of statistical analysis. [file 1741-7007-8-58-S9.pdf]

Supplementary Information for Witten, Tibshirani, Gu, Fire and Lui (2010)

1 Overview

We present a method for the identification of differentially-expressed miRNAs between tumor and normal classes, and we compare this method to the simple t-statistic scores that are often used for microarray data.

T-Statistic Approach

1. Take cube roots of the data.
2. Divide each patient's miRNA measurements by the mean miRNA counts for that patient. This is done in order to account for the large variation in total miRNAs between different patients.
3. Using the scaled data from the previous step, perform a paired two-sample t-test for each miRNA. The resulting t-statistics are treated as scores for each miRNA. (In fact, rather than ordinary t-statistics, modified t-statistics can be used, as is done in the Significance Analysis of Microarrays package. The modified t-statistics are obtained by adding a small constant to the denominator of the ordinary t-statistic.)
4. The t-statistics are used to form a ranked list of the most significant genes, and the false discovery rate of this list is estimated by data permutations.

Our new approach is as follows:

Log-Linear Model Approach

1. Take cube roots of the data.
2. Fit a Poisson log linear model to the data, allowing a separate term for each miRNA and each patient, as well as a term for each miRNA that captures the difference in average expression between tumor and normal samples. Call the latter term ρ_i for miRNA i.
3. The differential-expression score for miRNA i is $t_i = \frac{\rho_i}{se(\rho_i)}$, where $se(\rho_i)$ is the standard error of ρ_i. A high $|t_i|$ score indicates that the miRNA is differentially-expressed between tumor and normal samples.

4. The t_i scores are used to form a ranked list of the most significant genes, and the false discovery rate of this list is estimated by data permutations.

We also briefly present an overview of false discovery rate estimation. For a given miRNA score cut-off, the false discovery rate (FDR) is the average number of miRNAs with scores that are at or above the cut-off that are not truly differentially-expressed between tumor and normal samples. If, at a given score cut-off, the FDR is 0.2, then that means that we expect that 20% of the miRNAs with scores that exceed this cut-off are, in fact, false positives. We estimate false discovery rates as follows (for both the t-statistic approach and our proposed log-linear model):

False Discovery Rate Estimation

1. Let $(\mathbf{X}, \mathbf{y})$ denote our data, where the matrix $\mathbf{X}$ is the miRNA count data, and $\mathbf{y}$ is the list of labels for each of the 58 samples. Compute the scores of interest for each miRNA; let $t_i(\mathbf{X}, \mathbf{y})$ denote the score for miRNA i.

2. Create 500 new datasets of the form $(\mathbf{X}, \mathbf{y}^*)$, where $\mathbf{y}^*$ is obtained by shuffling the tumor/normal labels for each patient at random. Note that none of the miRNAs in these new data sets are differentially-expressed between "tumor" and "normal", because the tumor and normal class labels were created at random.

3. Compute the average number of miRNAs, across all 500 new data sets, with scores $|t_i(\mathbf{X}, \mathbf{y}^*)|$ that exceed the cut-off interest; call this quantity A_{null}.

4. Compute the average number of miRNAs in the real data set with scores $|t_i(\mathbf{X}, \mathbf{y})|$ that exceed the cut-off of interest; call this quantity A_{actual}.

5. For this cut-off value, the estimated false discovery rate is $\frac{A_{null}}{A_{actual}}$.

2 Log-Linear Model

Here, we present in detail the log-linear model mentioned in the previous section. Let $\mathbf{X}$ denote the 714×58 matrix of data, where the rows are the miRNAs and the columns are the patients. Let $\mathbf{Y}$ be the matrix of cube rooted data, $Y_{ij} = 1 + X_{ij}^{1/3}$.

The model is as follows:

$$
\begin{aligned}
Y_{ij} &\sim Poisson(\mu_{ij}) \\
\log \mu_{ij} &= \beta_i + \gamma_j + \rho_i(1_{j \in tumor} - 1_{j \in normal})
\end{aligned}
$$

for $i \in \{1, ..., 714\}$ and for $j \in \{1, ..., 58\}$, where i indexes the miRNAs (rows) and j indexes the patient samples. Here, $1_{j \in tumor}$ is an indicator variable that equals 1 if sample j corresponds to a tumor, and equals 0 otherwise. We fit the model in two steps:

1. We fit the following model:

$$\begin{aligned} Y_{ij} &\sim Poisson(\mu_{ij}) \\ \log \mu_{ij} &= \beta_i + \gamma_j \end{aligned}$$

   Let $\hat{\beta}_i$ and $\hat{\gamma}_j$ denote the coefficients obtained by fitting this model.

2. Fixing the coefficients $\hat{\beta}_i$ and $\hat{\gamma}_j$ obtained in the previous step, we fit the following model:

$$\begin{aligned} Y_{ij} &\sim Poisson(\mu_{ij}) \\ \log \mu_{ij} &= \hat{\beta}_i + \hat{\gamma}_j + \rho_i(1_{j \in tumor} - 1_{j \in normal}) \end{aligned}$$

So, our approach involves fitting two log linear models. We are modeling the counts for each miRNA for each patient as Poisson random variables, and we are estimating the logs of the means of these Poisson variables using a linear model. In this linear model, we allow a separate term (β_i) for each miRNA (since different miRNAs have different frequencies) and for each sample (γ_j) (since some samples have much higher levels of all miRNAs). The ρ_i term allows each miRNA to have different base levels between tumor and normal tissue.

3 Results

3.1 Model Fit

In this section, we investigate how well the model in Section 2 fits the data.

Under the Poisson model $Y_{ij} \sim Poisson(\mu_{ij})$, it follows that $E(Y_{ij}) = Var(Y_{ij}) = \mu_{ij}$. To see whether this holds for our data and the fitted model, we binned the 714×58 observations based on their value of $\hat{\mu}_{ij}$, and we estimated the mean and variance of the observations in each bin. The results can be seen in panel (a) of Figure 1. The mean and variance of the observations within each bin are approximately equal, and they also are nearly equal to the mean value of $\hat{\mu}_{ij}$ within each bin.

In order to see how closely the estimate for μ_{ij} fits the data, we can also make a scatterplot of $\hat{\mu}_{ij}$ against Y_{ij} for all i and j. The results are shown in Figure 1, panel (b). The points roughly follow the 45 degree line, which is expected if the model fits well.

Panel (c) of Figure 1 shows a histogram of the scaled residuals. They are approximately symmetric around zero.

Note that these analyses were also performed after the model in Section 2 was fit to the raw (rather than the cube rooted) data. The model fits the raw data extremely

poorly.

Figure 2 shows that the column means of the cube rooted data are roughly proportional to the sample-specific terms in the model proposed in Section 2. In other words, the standardization for each sample that we perform using our proposed model is not drastically different from the standardization that is performed using the t-statistic approach.

3.2 Identification of Significant miRNAs

To assess the "significance", or extent of differential expression, of each miRNA, we use the ρ_i terms obtained by fitting the model in Section 2. The score for miRNA i is $t_i = \frac{\rho_i}{se(\rho_i)}$, where the denominator is estimated by bootstrapping tumor/normal pairs.

Note that the scores obtained using the log linear model that we propose in Section 2 are not drastically different from those obtained using the standard t-statistic approach, as shown in Figure 3.

False discovery rates were estimated as discussed previously, and the results are given in the main paper.

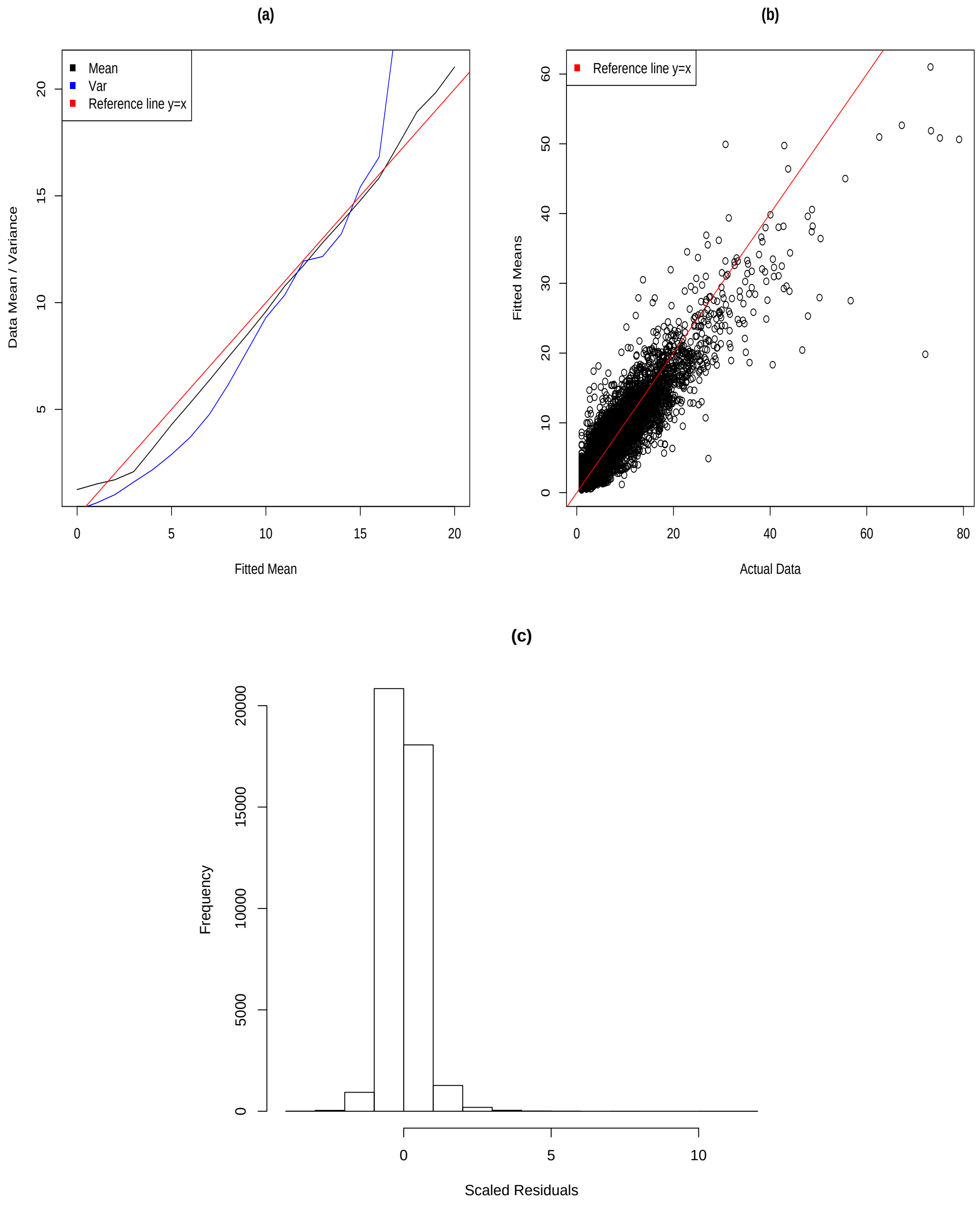

Figure 1: **Panel (a)**: Mean and variance of observed data, binned by value of $\hat{\mu}_{ij}$. Mean and variance are approximately equal, as expected under Poisson model. **Panel (b)**: Y_{ij} vs. $\hat{\mu}_{ij}$; the points lie approximately on the 45-degree line. **Panel (c)**: Histogram of the scaled residuals from the Poisson model. Residuals are roughly symmetric around zero.

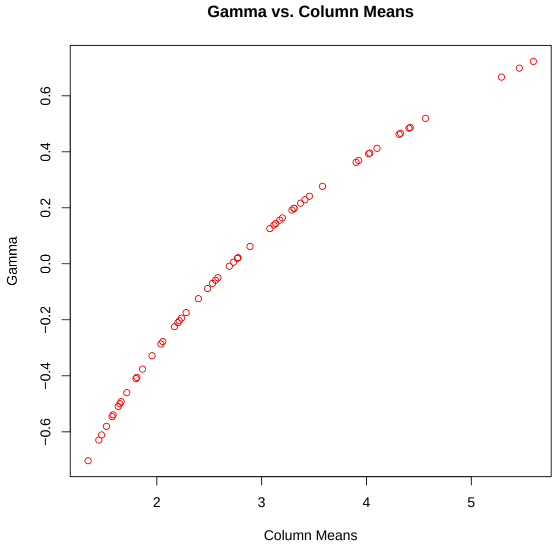

Figure 2: We compare the column means for the cube rooted data to the γ_j terms obtained in the model in Section 2. They are roughly proportional to each other. This means that scaling the cube rooted samples by their means is roughly equivalent to removing the sample-specific effects via a log linear model.

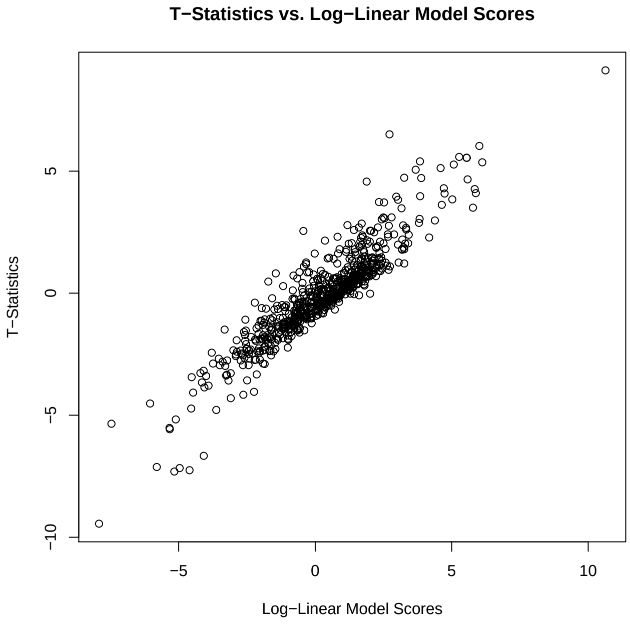

Figure 3: Scores obtained using the log linear model are highly correlated with those obtained using t-statistics.
