# Supplementary material for: Ultra-high throughput sequencing-based small RNA discovery and discrete statistical biomarker analysis in a collection of cervical tumours and matched controls
Source: BMC Biol. 2010 May 11;8:58. doi: 10.1186/1741-7007-8-58 (PMC2880020; doi:10.1186/1741-7007-8-58)
Supplement: Additional file 14 — Comparison of sequencing and Northern data of the 29 cervical cancer samples studied. [file 1741-7007-8-58-S14.doc]

|  | Northern blot analysis*^,1^ | | | Sequencing data^2^ | | |
| --- | --- | --- | --- | --- | --- | --- |
| Sample ID | *miR-21* |  | *miR-143* | *miR-21* |  | *miR-143* |
| G547 | ↑↑↑ |  | ↓↓↓ | 17.11 |  | 0.13 |
| G659 | ↑ |  | ↓↓ | 3.87 |  | 0.05 |
| G691 | ↑↑ |  | ↓↓ | 0.99 |  | 0.03 |
| G696 | ↑↑↑ |  | ↓↓ | 3.62 |  | 0.14 |
| G761 | ↑ |  | ↓↓ | 0.91 |  | 0.15 |
| G220 | ↑↑↑ |  | ND | 14.70 |  | 0.17 |
| G871 | NC |  | ↓↓ | 0.45 |  | 0.26 |
| G701 | ND |  | ↓↓↓ | 4.19 |  | 0.50 |
| G576 | ND |  | ND | 20.84 |  | 0.05 |
| G652 | ↑↑↑ |  | ↓↓↓ | 8.31 |  | 0.07 |
| G699 | ↑↑↑ |  | ↓↓↓ | 26.47 |  | 0.02 |
| G850 | ND |  | ND | 4.43 |  | 0.30 |
| G013 | ↑ |  | ↓↓↓ | 1.67 |  | 0.16 |
| G603 | ↑↑↑ |  | ↓↓ | 15.17 |  | 0.10 |
| G026 | ND |  | ↑↑↑ | 110.31 |  | 0.14 |
| G575 | ND |  | ND | 3.18 |  | 0.11 |
| G613 | NC |  | ↓↓↓ | 5.04 |  | 0.06 |
| G702 | ND |  | NA | 1.12 |  | 0.08 |
| G622 | ↑↑ |  | ↓↓↓ | 1.61 |  | 0.04 |
| G645 | ↑↑ |  | ↓↓↓ | 0.19 |  | 0.55 |
| G529 | ↑↑↑ |  | ND | 15.02 |  | 0.70 |
| G648 | ↑↑↑ |  | ↓ | 3.60 |  | 0.11 |
| G601 | ↑↑↑ |  | ↑↑↑ | 4.74 |  | 0.25 |
| G727 | ↑ |  | ↓ | 7.13 |  | 0.11 |
| G001 | ↑ |  | ↓↓↓ | 2.31 |  | 0.18 |
| G243 | ND |  | ↓↓↓ | 3.70 |  | 0.06 |
| G531 | ↑↑↑ |  | ↓↓↓ | 4.80 |  | 0.07 |
| G612 | ↑↑ |  | ↓↓↓ | 1.67 |  | 0.17 |
| G428 | ↑↑↑ |  | ↓ | 15.24 |  | 0.06 |

**Additional file 14.** Comparison of sequencing and Northern data of the 29 cervical cancer samples studied

*Relative abundance level in tumor sample as compared to its normal tissue counterpart; ↑, increased; ↓, decreased; ND, not determined; NC, no change; NA, not available.

^1^The Northern data has been published in Lui *et al*., 2007. Cancer Res 67: 6031-6043.

^2^The values are ratios between the fractions of sequences from each pool of library in tumor samples as compared to its normal cervical tissues.
